# CMR RV size assessment should include RV to LV volume ratio

**DOI:** 10.1186/1532-429X-17-S1-P394

**Published:** 2015-02-03

**Authors:** Stephan P Altmayer, Nicolle Losada, Amit R Patel, Karima Addetia, Mardi Gomberg-Maitland, Paul R Forfia, Yuchi Han

**Affiliations:** 1 Cardiology, University of Pennsylvania, Philadelphia, PA, USA; 2 Drexel University, Philadelphia, PA, USA; 3 Cardiology, University of Chicago, Chicago, IL, USA; 4 Cardiology, Temple University, Philadelphia, PA, USA

## Background

The right ventricular (RV) size responds to many cardiopulmonary diseases characterized by chronic pressure and volume overload. Cardiovascular magnetic resonance (CMR) is considered the "gold-standard" for RV evaluation. The RV end-diastolic volume indexed to body surface area (RVEDVi) has been used for RV size assessment, but this parameter alone may not be sensitive enough to detect RV dilation due to its wide normal range and the lack of consideration of individual heart size. We sought to determine if the assessment of right to left ventricular end-diastolic volume ratio (RVEDV/LVEDV) in addition to RVEDVi increased the detection of RV dilation in the RV CMR analysis. The application of this ratio is investigated in a control and a Pulmonary Arterial Hypertension (PAH) population.

## Methods

Clinical CMR exams were performed on a 1.5T Siemens scanner (Avanto, Siemens Health Systems, Germany) or a 1.5 T Philips scanner (Achieva, Best, Netherland). CMR derived ventricular function and volumes were measured in a control group (n = 76) and in patients with pulmonary arterial hypertension (PAH) (n = 46) using QMASS (Medis, Leiden, The Netherlands). Different criteria for the detection of RV enlargement, including RVEDVi and RVEDV/LVEDV ratio, were evaluated in both groups.

## Results

The left and right ventricular volumes are shown in Table 1 for both control and PAH patients. The range for the RVEDV/LVEDV ratio in the normal population (mean ±2SD) was 0.93 - 1.27 in males and 0.89 - 1.29 in females (Table). When all control subjects are considered together, the mean ratio ±2SD was 0.92 - 1.28. Given its narrower distribution of normal values, the ratio of RVEDV over LVEDV better discriminated the RV size differences between control and PAH groups than the RVEDVi (Figure). Adding this ratio to RVEDVi detected RV enlargement in 19.6% PAH patients (RVEDV/LVEDV > 1.28) that were not identified by the RVEDVi alone (>102 ml/m^2^ for females and >114 ml/m^2^ for males).

**Table 1:** Comparison of ventricular measurements in the control and PAH groups.

	Male			Female		
	Control (38)	PAH (8)	p-value	Control (38)	PAH (38)	p-value

RVEDV (ml)	172.9 ±31.3	283.7 ±96.2	<0.001	129.3 ±29.8	220.7 ±93.7	<0.001

RVEDVi (ml/m2)	86 ±14.3	133.1 ±38.4	<0.001	73.4 ±14.3	133.3 ±67.4	<0.001

RVSV (ml)	91.5 ±17.3	94.2 ±21.6	0.70	76.6 ±14.6	75.6 ±19.2	0.80

RVEF (%)	53.6 ±4.1	34.1 ±8.9	<0.001	58.3 ±6.3	38.4 ±13.6	<0.001

LVEDV (ml)	156.1 ±27.3	170.3 ±36.3	0.21	121.1 ±19.8	127.9 ±30.6	0.25

LVEDVi (ml/m2)	77.6 ±12.3	80.6 ±15.1	0.55	69.3 ±10.6	76.1 ±21.0	0.08

LVSV (ml)	97.8 ±17.1	98.5 ±20.1	0.91	77.7 ±15	73.2 ±22.0	0.30

LVEF (%)	61.7 ±4.1	54.6 ±8.0	<0.001	64.6 ±4.7	58.9 ±7.5	<0.001

RVEDV/LVEDV	1.11 ±0.08	1.68 ±0.54	<0.001	1.09 ±0.1	1.77 ±0.67	<0.001

RVEDV, right ventricle end-diastolic volume; RVEDVi, right ventricle end-diastolic index; RVSV, right ventricle stroke volume; RVEF, right ventricle ejection fraction; LVEDV, left ventricle end-diatolic volume; LVEDVi, left ventricle end-diastolic index; LVSV, left ventricle stroke volume; LVEF, left ventricle ejection fraction.

**Figure 1:**
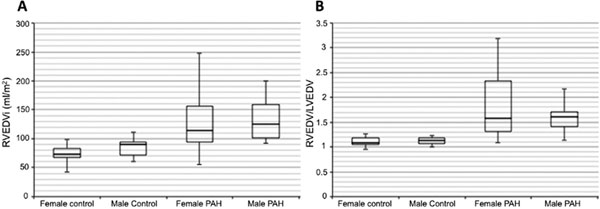


## Conclusions

The addition of the RVEDV/LVEDV ratio to RVEDVi increased the sensitivity of detection of RV enlargement in a PAH population.

## Funding

Cardiovascular Medical Research and Education Fund.

